# Outcomes in critically ill patients with cancer-related complications

**DOI:** 10.1186/2197-425X-3-S1-A251

**Published:** 2015-10-01

**Authors:** VBL Torres, JRL Vassalo, N Spector, FA Bozza, LCP Azevedo, JIF Salluh, M Soares

**Affiliations:** Internal Medicine, Universidade Federal do Rio de Janeiro, Rio de Janeiro, Brazil; D’Or Institute for Research and Education, Rio de Janeiro, Brazil; Hospital Sírio Libanês, São Paulo, Brazil

## Intr

The number of critically ill patients with malignancies has increased over the past decade and acute complications of cancer or its treatment are one of the most common causes to ICU admission.

## Objectives

Evaluate the clinical characteristics and outcomes of patients admitted to ICUs with complications related to cancer or its treatment in order to identify independent risk factors associated with mortality rates.

## Methods

Secondary analysis of two prospective Brazilians cohort studies. We used logistic regression to identify variables associated with hospital mortality.

## Results

Out of 2028 patients, 456 (23%) had at least one cancer-related complication at ICU admission. Compared to those without complications, they had worse performance status (PS) (57% vs 36% with PS ≥ 2, *P* < 0.001) and had more active disease (5% vs 43%, *P* < 0.001). The median SOFA score was higher [8 (5-11) vs 6 (4-9), *P* < 0.001], as well as the need for vasopressors, mechanical ventilation (MV) and dialysis (45% vs 34%, 70% vs 51% and 12% vs 8%, respectively) (*P* < 0.001 for all). The hospital length of stay (LOS) was similar in both subgroups (*P* = 0.501), but those with complications showed increased ICU mortality (47% vs 27%, *P* < 0.001). The most frequent complications were chemotherapy toxicity (5%), venous thromboembolism (5%), respiratory failure by tumor (RFBT) (4%), gastrointestinal complications by tumor (GCBT) (3%) and vena cava syndrome (VCS) (2%). 39/456 patients with complications received chemotherapy and/or radiotherapy at ICU, with no mortality difference. Adjusting for the type of admission, hospital LOS prior to ICU and patient's age, the variables independently associated with hospital mortality were: PS≥2 [OR = 2.56 (2.05-3.20), *P* < 0.001], metastatic solid tumor [OR = 2.11 (1.59-2.80), *P* < 0.001], high-grade hematologic malignancy [OR = 2.08 (1.36-3.17), *P* = 0.001], higher SOFA score [OR = 1.16 (1.13-1.20), *P* < 0.001], MV [OR = 4.08 (3.23 - 5.15), *P* < 0.001], *P <* 0.001], presence of VCS [OR = 3.72 (1.10 - 12.58), *P* = 0.035], GCBT [OR = 2.56 (1.29-5.09), *P* = 0.007] and RFBT [OR = 1.97(1.04-3.71), *P* = 0.37].

## Conclusions

The prognostic impact of cancer-related complications is variable. The presence a severe acute cancer-related complication *per se* should not guide decisions to admit a patient to the ICU. However, patients presenting with VCS, GCBT and RFBT had worse outcomes.
